# Blood and local eosinophil levels in chronic rhinitis: Observations during seasonal allergen exposure and non-exposure

**DOI:** 10.1016/j.waojou.2024.100930

**Published:** 2024-07-04

**Authors:** Xu Xu, Jingyun Li, Xu Zhang, Lin Xi, Yunbo Gao, Xian Li, Yuan Zhang, Luo Zhang

**Affiliations:** a Department of Allergy, Beijing Tongren Hospital, Capital Medical University, Beijing 100730, China; b Department of Otolaryngology Head and Neck Surgery, Beijing Tongren Hospital, Capital Medical University, Beijing 100730, China; c Beijing Laboratory of Allergic Diseases and Beijing Key Laboratory of Nasal Diseases, Beijing Institute of Otolaryngology, Beijing 100005, China; d Beijing Key Laboratory of Nasal Diseases, Beijing Institute of Otolaryngology, Beijing 100005, China

**Keywords:** Allergen exposure, Allergic rhinitis, Chronic rhinitis, Eosinophil

## Abstract

**Background:**

Allergic rhinitis (AR) is a typical type 2 inflammatory disease and eosinophils play a critical role of cardinal effectors in type 2 inflammation. However, the distribution of eosinophils in patients with different subtypes of rhinitis and the effect of allergen exposure on them remain understudied. The aim of this study was to investigate the characteristics and factors influencing the distribution of systemic and local eosinophils in patients with non-AR (NAR), perennial AR (PAR), and seasonal AR (SAR), as well as the effect of seasonal allergen exposure levels on eosinophils.

**Methods:**

This was a population-based, cross-sectional observational study of consecutive chronic rhinitis (CR) outpatients who volunteered to participate in the survey during the spring pollen season and non-pollen season of 2023 in Beijing. All participants underwent serum allergen testing, blood routine examination, and nasal secretion smear cytology, and completed questionnaires mainly involving basic information, history review, and symptom assessment. Spring pollen dispersal concentration were considered.

**Results:**

A total of 558 CR patients eligible for enrollment were collected, including 198 NAR, 204 PAR, and 156 SAR patients. PAR had the highest blood eosinophil levels and the most severe overall nasal and ocular symptoms of SAR. Compared with subjects with blood eosinophil counts <0.3 × 10^9^/L, those with ≥0.3 × 10^9^/L had significantly more severe nasal and ocular symptoms and a significantly higher rate of comorbid asthma and allergic conjunctivitis. Blood eosinophil levels were significantly higher in SAR patients during the pollen season compared to the non-pollen season, and pollen concentrations were positively correlated with systemic and local eosinophil levels.

**Conclusions:**

Blood eosinophil levels varied in patients with different subtypes of rhinitis. Patients with high blood eosinophil levels had more severe overall nasal and ocular symptoms, and that blood eosinophils levels were significantly higher in patients with asthma or allergic conjunctivitis than patients without comorbidities. There was a positive trend between allergen exposure and systemic and local eosinophil levels. Further longitudinal cohort studies are still needed to better analyze the influence of eosinophil levels on the clinical traits of AR.

## Introduction

Chronic rhinitis (CR) is a common inflammatory disease of the nasal mucosa characterized by nasal congestion, nasal itching, sneezing, and rhinorrhea. Clinically, CR is classified into allergic rhinitis (AR) and non-allergic rhinitis (NAR) based on its etiology. Based on the type of allergen, AR is categorized into perennial AR (PAR) and seasonal AR (SAR), where SAR is mainly caused by pollen transmission. AR is among the most prevalent diseases globally, with the prevalence of self-reported AR estimated to range from approximately 2%–25% in children and from 1% to over 40% in adults.^1^ The overall prevalence of AR in different cities in China ranges from 10 to 24% and has increased dramatically in recent years.^2^^,^^3^ Although AR is not a life-threatening disease, its high prevalence and nasal symptoms can have a significant impact on work productivity.^4^ In Beijing, the total cost of AR patients is €195.6 person/year, with a total social cost of about €440.9 million per year, which implies a huge socio-economic burden.^5^

Pathophysiologically, AR is an IgE-mediated response, type 2 inflammatory disease.^6^ Indeed, eosinophils play an important role in the development of allergic inflammation in airway and skin tissues as a potential end-stage effector and blood immune biomarker.^7^ Eosinophils mature in the bone marrow and was released into the peripheral blood and enter tissues in response to cooperative signaling by IL-5 and eotaxin family chemokines.^8^ Studies have shown that blood eosinophil levels are significantly elevated in AR patients and correlate with the severity of their symptoms.^9^^,^^10^ Yenigun and colleagues have reported that the eosinophil-to-lymphocyte ratio was significantly elevated in children with AR and could be used for the diagnosis and clinical follow-up of pediatric AR patients.^11^ Early life aeroallergen sensitization and elevated blood eosinophils are robust predictors of asthma development.^12^ A multicenter longitudinal study in China reported high blood eosinophil count as a risk factor for developing asthma.^13^ The possible biological mechanism is that eosinophils cause tissue damage in inflammation by synthesizing and releasing a variety of specific cytotoxic effector proteins.^14^ Eosinophil depletion in humans is clinically beneficial in a number of diseases, and ‘‘human eosinophil knockouts,’’ generated by these drugs, do not demonstrate any major deficits, and many cutting-edge technologies are currently being leveraged to uncover mechanisms of eosinophilic diseases and are poised to identify novel therapeutic targets to deplete and regulate eosinophils in health and disease.^15^

So far, a large number of studies have confirmed the correlation between pollen concentration or blood eosinophil levels and symptom severity in AR patients, but the relationship between pollen exposure and blood and local eosinophil levels remains unknown. Based on these considerations, we collected data from a cohort of CR patients who presented to the clinic with nasal and ocular symptoms and subdivided them according to allergens into NAR, PAR, and SAR to spring pollen allergy, with the aim of evaluating the differences in systemic and local eosinophil levels and their association with clinical features, analyzing the characteristics of eosinophilic inflammation in AR, as well as assessing the effect of spring pollen exposure on systemic and local eosinophil levels. Moreover, we explored the concordance between invasive and noninvasive eosinophils assays in terms of response potency.

## Methods

### Study design

This was a population-based, cross-sectional observational study of consecutive CR patients seen from February 1, 2023 to July 31, 2023 for nasal and ocular symptoms. Pollen season is defined as the first day when the total daily pollen count was ≥80 grains/1000 mm^2^ to the first day when the total daily pollen count was <80 grains/1000 mm^2^.^16^ Therefore, based on broadcast data from the Beijing Meteorological Bureau (https://m.weather.com.cn/huafen/index.html?id=101010100) on total pollen concentrations in the environment, the spring pollen season was defined as March 5, 2023 to May 29, 2023, and the remaining time was defined as the non-pollen season. All participants underwent a serum allergen testing, a blood routine examination, and a nasal secretion smear cytology on the day of visit, and completed a questionnaire.

The study protocol was approved by the ethics review board and written informed consent was obtained from all participants before enrolment into the study.

### Subjects and diagnosis

Patients were eligible to join the study if they met the following criteria: (1) age≥14 years; (2) permanent population in Beijing area (resident for more than half a year); (3) had 2 or more typical nasal symptoms in the past year, including nasal congestion, nasal itching, sneezing and rhinorrhea; (4) the diagnosis of AR was based on the international diagnostic criteria of AR guidelines (Allergic Rhinitis and its Impact on Asthma, ARIA),^1^ with (a) pale and edematous nasal mucosa and watery nasal secretions, (b) positive allergen test, (c) without CRS, NP or other nasal tumors; (5) the diagnosis of asthma was based on the Global Initiative for Asthma (GINA) guidelines^17^ and was characterized by (a) variable wheezing, tightness, chest tightness, cough, (b) objective evidence of variable airflow limitation; (6) the diagnosis of allergic conjunctivitis (AC) was based on the Consensus of Experts on the Diagnosis and Treatment of Allergic Conjunctivitis (2018),^18^ and was characterized by (a) ocular itching accompanied by foreign body sensation and increased conjunctival sac secretions, (b) conjunctival congestion, conjunctival papilla, and corneal specific lesions of at least 1 item; (7) the diagnosis of atopic dermatitis (AD) was based on the Williams criteria, which focus on skin itching.^19^ Participants were categorized into NAR, PAR, and SAR based on allergen testing results. NAR was defined as negative for all allergens tested. PAR was defined as sensitization to perennial allergens only, while SAR was only allergic to spring allergens. To avoid the influence of mixed allergens on the study, we excluded patients who were sensitized to both seasonal and perennial allergens.

Based on the reported literature, the blood eosinophil count in patients with persistent allergic rhinitis and intermittent allergic rhinitis was (339 ± 246) × 10^3^/μl and (232 ± 195) × 10^3^/μl, respectively.^10^ The α was set at 0.05 and 1-β was set at 0.8. The calculated number of people was 138. Set a sample attrition rate of 10%, the total number of people was 152. Therefore, in this study, 198 people were included in NAR, PAR participants were 204, and SAR were 156.

### Serum IgE antibodies

Serum specific IgE (sIgE) levels to common airborne allergens were determined using the Immunoblotting (AllergyScreen™, German), with a value for serum sIgE ≥0.35 kUA/l regarded as positive. We tested a total of 17 airborne allergen indicators, of which 3 were mixed allergens, and finally covered 32 allergens. These included 9 perennial allergen indicators (*Dermatophagoides pteronyssinus*, *Dermatophagoides farina*, *Blomia tropicalis*, cat dander, dog dander, cockroach, *Aspergillus fumigatus*, fungi group [candida, *Penicillium notatum*, *Cladosporium*, *Alternaria* spp, *Aspergillus niger*] and silk), 4 spring allergen indicators (juniper/birch, *Platanaceae/Fraxinus chinensis*, spring pollen group 1 [poplar, willow, beech, oak, walnut], and spring pollen group 2 [ashleaf maple, mulberry, false acacia, elm, cypress, kozo]), and 4 autumn allergen indicators (mugwort, ragweed, japanese hop and goosefoot/*Amaranthus retroflexus*). Serum total IgE level was determined using ImmunoCAP system (Pharmacia, Uppsala, Sweden).

### Blood eosinophils

Routine blood tests were performed on all participants to detect systemic levels of eosinophil. Chen et al found that the eosinophil levels in the blood were significantly higher in mild and moderate to severe AR compared to healthy controls, and the moderate to severe patients had higher levels of blood eosinophils than the mild patients, with a mean blood eosinophil count of ≥0.3 × 10^9^/L.^9^ Based on the relationship between AR patients blood eosinophil levels and clinical symptom severity, we categorized all participants into low-eosinophil (blood eosinophil count <0.3 × 10^9^/L) and high-eosinophil (blood eosinophil count ≥0.3 × 10^9^/L) groups.

### Local eosinophils

All participants underwent nasal secretion smear cytology, an easy and noninvasive test in which a specimen of secretion is obtained by scraping the meatus nasi medius and the anterior end of the inferior turbinate with a cotton swab. After staining by Eosin-Meilan method, the smears were observed under an optical microscope with a 10x eyepiece and a 40× objective. Eosinophils were divided into 6 classes: “-” means none; “±” means occasional; “+” means few scattered cells, small clumps; “++” means moderate number, large clumps; “+++” means large clumps not covering the field; “++++” means clumps covering entire field.^20^ In this study, due to the small number of “±” groups, we categorized “±” as “+” groups for statistical convenience.

### Questionnaire design

The questionnaire consisted of 3 sections: basic information, history review, and symptom assessment, which provided information on the participant's general demographic characteristics (age, gender, current city of residence, etc), as well as the duration of rhinitis, self-reported allergens, self-reported other allergic diseases (asthma, allergic conjunctivitis, and atopic dermatitis), and a history of smoking, drinking, and medication use in the last week. The severity of typical nasal and ocular symptoms (nasal congestion, nasal itching, sneezing, rhinorrhea, ocular pruritus/redness, and lacrimation) was assessed by validated Chinese version visual analogue scale (VAS).

### Symptom assessments

In addition to individual symptom VAS scores, we used total nasal symptoms (TNSS), total ocular symptoms (TOSS), and average total combined score (ATCS) to evaluate the severity of patients' symptoms. The VAS scores were converted to 0–3 based on the conversion criteria of 0.1–3 as mild, 3.1–7 as moderate, and 7.1–10 as severe.^21^ TNSS (range: 0–12) and TOSS (range: 0–6) were the sum of the scores of 4 nasal symptoms and 2 ocular symptoms, respectively. Furthermore, considering the impact of medication use on patients' clinical symptoms, we set up a symptomatic drug combination evaluation index. The ATCS was defined as the sum of the total symptom score (range: 0–18) and the medication score (range: 0–3; 0 = no treatment, 1 = only an oral antihistamine, 2 = an intranasal corticosteroid with/without oral antihistamine, 3 = an oral corticosteroid with/without oral antihistamine, with/without intranasal corticosteroid).^22^

### Statistical analysis

Kruskal-Wallis test and chi-square test were used for comparison of clinical parameters between groups and Mann-Whitney *U* test was used for comparison between 2 groups. Spearman correlation analysis was used to assess the linear relationship between the variables. All the statistics between groups were performed by using SPSS version 26.0 (IBM Corp., Armonk, NY, USA) and GraphPad Prism 8.0 software (GraphPad, Inc., La Jolla, CA, USA). Data were expressed as percentages and interquartile ranges. Statistical significance was set at a *P* value of <0.05.

## Results

### Demographic and clinical characteristics

A total of 558 patients aged 14 years and older with nasal and ocular symptoms were collected during the spring pollen and non-pollen seasons in 2023, including 198 in the NAR group, 204 in the PAR group, and 156 in the SAR group (Table 1). The results showed a higher proportion of older patients in the NAR group compared to the other groups. The SAR population had the highest rate of comorbid allergic conjunctivitis and had the most severe 2 ocular symptoms, total nasal and ocular symptoms, ATCS scores, and the highest serum total IgE levels. We analyzed the differences in blood eosinophil count, percentage, eosinophil-neutrophil ratio (ENR), and eosinophil-lymphocyte ratio (ELR) among the 3 populations at the same time, and the highest levels of blood eosinophils were found in the PAR population. No differences in local eosinophil levels were seen among the 3 groups.

**Table 1:** Demographic and clinical characteristics of the study population.

	NAR (N = 198)	PAR (N = 204)	SAR (N = 156)	*P* value
Male, n (%)	95 (48.0%)	93 (45.6%)	82 (52.6%)	0.418
Age (years)	36.00 (31.00–42.25)	33.00 (29.00–39.75)	34.00 (28.00–40.00)	**0.002**^a^^,^^b^
Disease duration (years)	3.50 (1.75–8.00)	4.00 (1.40–9.38)	5.00 (2.00–10.00)	0.246
Smoking, n (%)	32 (16.2%)	34 (16.7%)	24 (15.4%)	0.948
Comorbidity, n (%)				
Asthma	11 (5.6%)	23 (11.3%)	12 (7.7%)	0.109
Allergic conjunctivitis	16 (8.1%)	39 (19.1%)	41 (26.3%)	**<0.001**^a^^,^^b^
Allergic dermatitis	35 (17.7%)	44 (21.6%)	24 (15.4%)	0.306
Symptom score				
Nasal congestion	6.05 (3.18–8.20)	7.00 (3.93–8.98)	6.70 (4.00–8.50)	0.184
Nasal itching	5.10 (2.25–7.63)	5.70 (3.00–7.68)	6.40 (3.00–8.00)	0.176
Sneezing	6.90 (4.40–8.75)	6.60 (5.00–8.68)	7.00 (4.33–8.60)	0.678
Rhinorrhea	6.90 (3.88–8.83)	7.00 (4.75–9.00)	7.10 (4.45–8.98)	0.372
Ocular pruritus/redness	3.00 (0.20–6.90)	3.10 (0.50–7.00)	5.00 (1.48–8.60)	**<0.001**^b^^,^^c^
Lacrimation	1.00 (0.00–5.00)	1.85 (0.00–5.08)	2.30 (0.40–5.93)	**0.031**^b^
Drug score	1.00 (0.00–2.00)	1.00 (0.00–2.00)	1.00 (0.00–2.00)	0.978
TNSS	8.00 (6.00–11.00)	9.00 (8.00–10.00)	9.00 (7.00–11.00)	0.208
TNSS+TOSS	11.00 (8.00–14.00)	11.00 (9.00–14.00)	12.00 (9.00–15.00)	**0.018**^b^
ATCS	12.00 (9.00–15.00)	12.00 (10.00–15.00)	13.00 (10.00–16.00)	**0.040**^b^
Serum total IgE (kU/L)	41.25 (20.90–79.43)	118.50 (51.50–272.25)	146.00 (65.70–319.00)	**<0.001**^a^^,^^b^
Blood eosinophil count (×10^9^/L)	0.22 (0.12–0.36)	0.30 (0.14–0.48)	0.27 (0.14–0.40)	**0.005**^a^
Blood eosinophil %	3.40 (1.90–5.40)	4.60 (2.32–7.00)	3.90 (2.40–6.07)	**0.004**^a^
ENR	0.06 (0.03–0.10)	0.08 (0.04–0.14)	0.07 (0.04–0.10)	**0.002**^a^
ELR	0.11 (0.06–0.17)	0.15 (0.08–0.22)	0.12 (0.07–0.19)	**0.005**^a^
Nasal secretion eosinophils (−/+/++/+++/++++)	20.0%/22.1%/23.6% /28.6%/5.7%	11.9%/22.2%/27.4% /25.2%/13.3%	15.2%/19.0%/32.4% /21.9%/11.4%	0.211

NAR, nonallergic rhinitis; PAR, perennial allergic rhinitis; SAR, seasonal allergic rhinitis; TNSS, total nasal symptoms; TOSS, total ocular symptoms; ATCS, average total combined score; ENR, eosinophil-neutrophil ratio; ELR, eosinophil-lymphocyte ratio.

a There was a significant difference between NAR and PAR.

b There was a significant difference between NAR and SAR.

c There was a significant difference between PAR and SAR

### Relationship between blood eosinophil levels and clinical characteristics

All participants were divided into low and high level groups by whether the blood eosinophil count was <0.3 × 10^9^/L. Table 2 demonstrated the differences in clinical characteristics between the 2 populations. Patients in the low-level group were older and had a longer duration of illness. Patients in the high-level group had more severe nasal and ocular symptoms and higher serum total IgE levels. Trends in local eosinophil count levels were consistent with blood eosinophil count levels. Notably, we observed that the proportion of patients with comorbid asthma in the high-level group was nearly twice as high as in the low-level group (11.0% vs. 6.2%, *P* = 0.041). Although not statistically different, the proportion of combined allergic conjunctivitis was also higher in the high-level group (20.8%) than in the low-level group (14.6%). Based on this, we separately analyzed the effect of comorbid asthma, allergic conjunctivitis, and atopic dermatitis on eosinophil levels (Table 3). The results showed that patients with comorbid asthma/allergic conjunctivitis had significantly higher blood eosinophil count, blood eosinophil percentage, and ENR than patients without comorbid asthma/allergic conjunctivitis (patients with comorbid asthma also had higher levels of ELR). However, no differences were seen in comparing patients with and without atopic dermatitis.

**Table 2:** Clinical characterization of high and low levels of blood eosinophil.

	Low-blood eosinophil (N = 322)	High-blood eosinophil (N = 236)	*P* value
Male, n (%)	156 (48.4%)	114 (48.3%)	0.974
Age (years)	35.00 (31.00–41.00)	33.00 (29.00–40.00)	**0.018**
Disease duration (year)	4.15 (2.00–10.00)	3.23 (1.25–8.00)	**0.037**
Smoking, n (%)	46 (14.3%)	44 (18.6%)	0.167
Comorbidity, n (%)			
Asthma	20 (6.2%)	26 (11.0%)	**0.041**
Allergic conjunctivitis	47 (14.6%%)	49 (20.8%)	0.057
Allergic dermatitis	59 (18.3%)	44 (18.6%)	0.923
Symptom score			
Nasal congestion	5.65 (2.48–8.00)	7.60 (4.93–9.00)	**<0.001**
Nasal itching	5.20 (2.40–7.50)	6.00 (3.20–8.00)	**0.042**
Sneezing	6.50 (3.98–8.43)	7.00 (5.00–8.80)	**0.030**
Rhinorrhea	6.95 (3.40–8.80)	7.60 (5.00–9.00)	**0.004**
Ocular pruritus/redness	3.00 (0.30–6.93)	5.10 (1.03–8.00)	**<0.001**
Lacrimation	1.05 (0.00–5.00)	2.30 (0.23–6.00)	**<0.001**
Drug score	1.00 (0.00–2.00)	1.00 (0.00–2.00)	0.340
TNSS	8.00 (7.00–10.00)	9.00 (8.00–11.00)	**<0.001**
TNSS+TOSS	11.00 (8.00–14.00)	12.00 (10.00–15.00)	**<0.001**
ATCS	12.00 (9.00–15.00)	13.00 (11.00–16.00)	**<0.001**
Serum total IgE (kU/L)	67.85 (27.55–170.25)	116.00 (44.70–318.00)	**<0.001**
Nasal secretion eosinophils (−/+/++/+++/++++)	20.8%/25.0%/28.8% /21.2%/4.2%	9.5%/16.7%/25.6% /31.0%/17.3%	**<0.001**

Low-blood eosinophil, blood eosinophil count <0.3 × 10^9^/L; High-blood eosinophil, blood eosinophil count ≥0.3 × 10^9^/L; TNSS, total nasal symptoms; TOSS, total ocular symptoms; ATCS, average total combined score

**Table 3:** Effect of comorbidity with other allergic diseases on eosinophil levels.

	without AS (N = 512)	with AS (N = 46)	*P* value
Blood eosinophil count (×10^9^/L)	0.26 (0.13–0.40)	0.33 (0.18–0.54)	**0.022**
Blood eosinophil %	3.90 (2.10–6.18)	4.85 (2.70–8.20)	**0.013**
ENR	0.07 (0.03–0.11)	0.09 (0.04–0.17)	**0.008**
ELR	0.12 (0.06–0.19)	0.18 (0.10–0.25)	**0.003**
Nasal secretion eosinophils (−/+/++/+++/++++)	14.7%/21.3%/28.2% /26.1%/9.8%	28.1%/21.9%/18.8% /18.8%/12.5%	0.199
	**without AC (N=462)**	**with AC (N=96)**	***P* value**
Blood eosinophil count (×10^9^/L)	0.26 (0.13–0.40)	0.30 (0.17–0.50)	**0.034**
Blood eosinophil %	3.75 (2.10–6.13)	4.70 (2.60–6.78)	**0.037**
ENR	0.07 (0.03–0.11)	0.08 (0.04–0.14)	**0.033**
ELR	0.12 (0.06–0.19)	0.15 (0.09–0.21)	0.059
Nasal secretion eosinophils (−/+/++/+++/++++)	16.9%/20.9%/27.9% /24.5%/9.8%	9.3%/24.1%/24.1% /31.5%/11.1%	0.237
	**without AD (N=455)**	**with AD (N=103)**	***P* value**
Blood eosinophil count (×10^9^/L)	0.26 (0.13–0.41)	0.28 (0.12–0.39)	0.961
Blood eosinophil %	4.00 (2.20–6.20)	4.10 (2.30–6.10)	0.904
ENR	0.07 (0.04–0.12)	0.07 (0.04–0.11)	0.902
ELR	0.13 (0.07–0.20)	0.13 (0.06–0.21)	0.660
Nasal secretion eosinophils (−/+/++/+++/++++)	15.6%/19.9%/27.0% /27.4%/10.1%	16.4%/27.4%/28.8% /17.8%/9.6%	0.179

AS, asthma; AC, allergic conjunctivitis; AD, atopic dermatitis; ENR, eosinophil-neutrophil ratio; ELR, eosinophil-lymphocyte ratio

### Effect of pollen exposure on eosinophil levels

Medications, especially hormonal drugs, can have an effect on eosinophil levels,^23^ so when exploring the relationship between pollen exposure and eosinophil levels, we only analyzed those with a drug score of 0 (Table 4). PAR populations did not differ in either systemic or local eosinophil levels during the pollen and non-pollen seasons. The proportion of local eosinophils "++" and above was significantly higher in the pollen phase than in the non-pollen phase of the NAR population (*P* = 0.001). In contrast, for the SAR population, blood eosinophil count, blood eosinophil percentage, ENR, and ELR levels were significantly higher during the pollen season compared to the non-pollen season (*P* < 0.05).

**Table 4:** Eosinophil levels between pollen and non-pollen seasons in NAR, PAR and SAR populations those with a drug score of 0.

	Non-pollen season	Pollen season	*P* value
NAR	N = 41	N = 45	
Blood eosinophil count (×10^9^/L)	0.16 (0.10–0.29)	0.22 (0.12–0.40)	0.261
Blood eosinophil %	2.50 (1.70–3.95)	3.10 (1.90–5.70)	0.088
ENR	0.04 (0.03–0.07)	0.05 (0.03–0.10)	0.130
ELR	0.09 (0.05–0.14)	0.12 (0.06–0.20)	0.133
Nasal secretion eosinophils (−/+/++/+++/++++)	29.6%/33.3%/22.2% /14.8%/0%	11.8%/5.9%/41.2% /32.4%/8.8%	**0.001**
PAR	N = 44	N = 51	
Blood eosinophil count (×10^9^/L)	0.32 (0.12–0.53)	0.26 (0.13–0.39)	0.462
Blood eosinophil %	4.80 (2.23–7.00)	4.20 (2.10–6.30)	0.446
ENR	0.09 (0.04–0.14)	0.08 (0.04–0.12)	0.638
ELR	0.16 (0.07–0.24)	0.14 (0.07–0.20)	0.506
Nasal secretion eosinophils (−/+/++/+++/++++)	10.7%/32.1%/28.6% /10.7%/17.9%	13.2%/13.2%/39.5% /26.3%/7.9%	0.551
SAR	N = 22	N = 46	
Blood eosinophil count (×10^9^/L)	0.14 (0.09–0.28)	0.28 (0.18–0.45)	**0.007**
Blood eosinophil %	2.55 (1.55–3.47)	4.10 (2.48–6.10)	**0.008**
ENR	0.04 (0.03–0.07)	0.07 (0.04–0.12)	**0.004**
ELR	0.08 (0.05–0.15)	0.12 (0.09–0.18)	**0.014**
Nasal secretion eosinophils (−/+/++/+++/++++)	20.0%/10.0%/50.0% /10.0%/10.0%	11.1%/13.9%/30.6% /27.8%/16.7%	0.283

NAR, nonallergic rhinitis; PAR, perennial allergic rhinitis; SAR, seasonal allergic rhinitis; ENR, eosinophil-neutrophil ratio; ELR, eosinophil-lymphocyte ratio

**Fig. 1:**
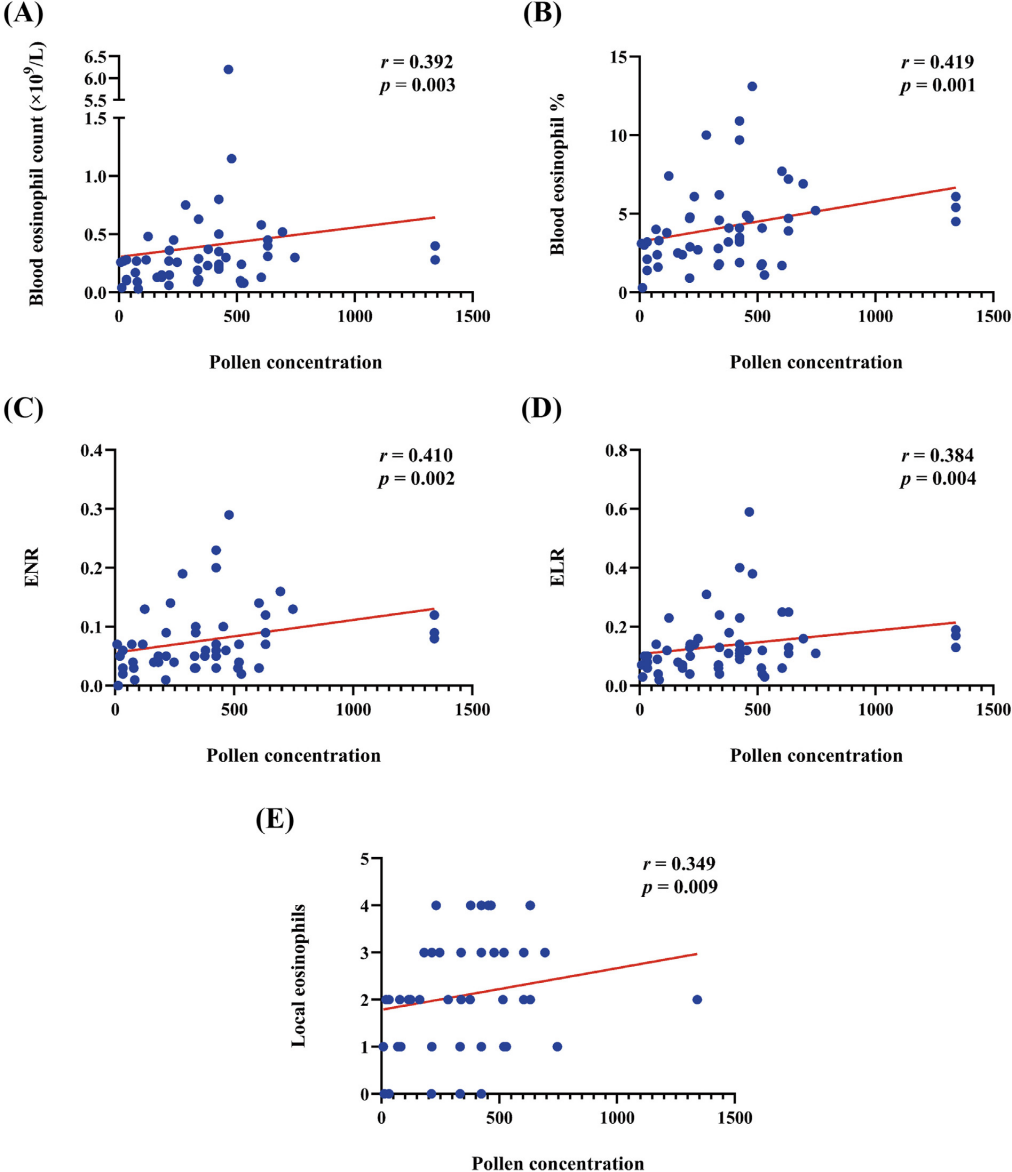
Correlation analysis between continuous three-day moving average pollen levels and eosinophil levels in the SAR population with a drug score of 0. Correlation between pollen concentration and (A) blood eosinophil count, (B) percentage of blood eosinophils, (C) ENR, (D) ELR and (E) local eosinophil levels. ENR, eosinophil-neutrophil ratio; ELR, eosinophil-lymphocyte ratio.

Subsequently, we further assessed associations between continuous three-day moving average pollen levels (average of today [lag 0], yesterday [lag 1], and 2 days ago [lag 2])^24^ and eosinophil levels in the SAR population with a drug score of 0 (Fig. 1). Blood eosinophil count (*r* = 0.392, *P* = 0.003), blood eosinophil percentage (*r* = 0.419, *P* = 0.001), ENR (*r* = 0.410, *P* = 0.002), ELR (*r* = 0.384, *P* = 0.004), and local eosinophil levels (*r* = 0.349, *P* = 0.009) were correspondingly elevated with the increased pollen concentration.

### Consistency of systemic and local eosinophil levels

**Fig. 2:**
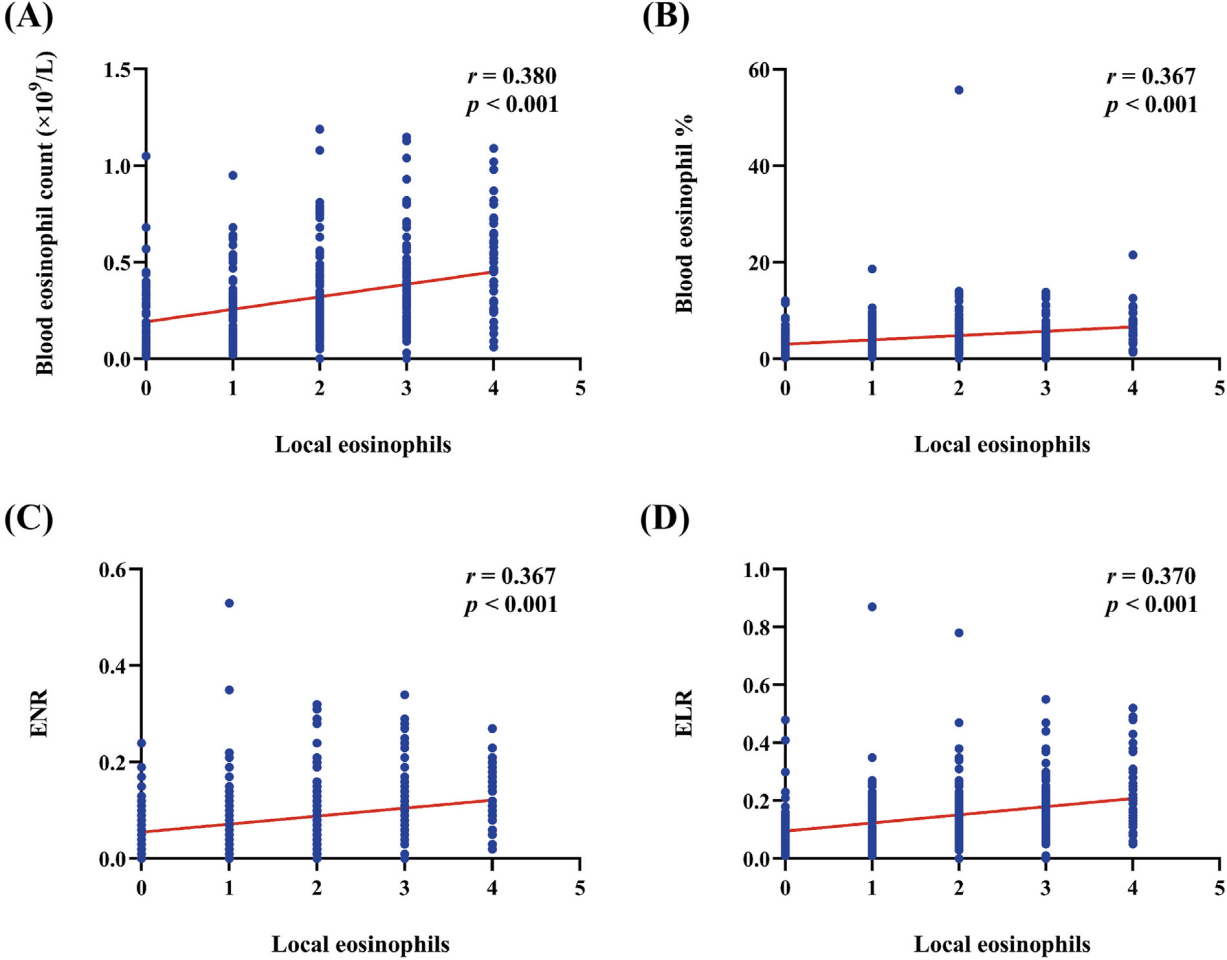
Correlation analysis of local and systemic eosinophil levels. Correlation between local eosinophil levels and (A) blood eosinophil count, (B) percentage of blood eosinophils, (C) ENR and (D) ELR. ENR, eosinophil-neutrophil ratio; ELR, eosinophil-lymphocyte ratio.

In the present study, we compared local and systemic eosinophil levels consistently and found a positive correlation between local trends and blood eosinophil indicators (blood eosinophil count [*r* = 0.380, *P* < 0.001], blood eosinophil percentage [*r* = 0.367, *P* < 0.001], ENR [*r* = 0.367, *P* < 0.001], ELR [*r* = 0.370, *P* < 0.001]) in the total population (Fig. 2).

## Discussion

Eosinophils are multifunctional leukocytes, being involved in the host defense against helminth infection, tissue homeostasis and repair of injured tissue, and also play critical roles in shaping the pathogenesis of allergic diseases.^25^ Although ever-increasing knowledge is gained on the function of eosinophils, it is still not known exactly when and how they relate to the development of allergic disease severity. Chen et al revealed the difference of the functional state of eosinophils in AR patients in relation to disease severity, with patients with moderate-severe AR were characterized by elevated levels of activated and pathogenic eosinophils, which were associated with higher production of eosinophilic cation protein (ECP), eosinophil peroxidase (EPX), and IL-4 in the peripheral blood.^9^ In this cross-sectional study, we focused on the characteristics of eosinophils distribution and its influencing factors in patients with different subtypes of rhinitis, as well as the effect of pollen exposure on the systemic and local eosinophil levels produced in AR patients.

Kant et al reported a more pronounced increase in absolute eosinophil, ENR and ELR levels in patients with persistent AR compared to intermittent AR.^10^ This is consistent with our results. In patients with PAR, due to the presence of perennial allergen stimulation, the nasal mucosa is in a state of long-term inflammatory reaction, and mucosal edema and hyperplasia are obvious, which is the main reason for the markedly elevated level of blood eosinophils and the significant symptoms of nasal congestion in the patients. In contrast, overall nasal and ocular symptoms, especially eye irritation, were significantly heavier in the SAR population exposed through the pollen saeson than in the NAR and PAR populations. It is worth noting that in the NAR population we need to further identify other diseases such as vasomotor rhinitis and nonallergic rhinitis with eosinophilia syndrome, which have similar main symptoms to AR but negative allergen tests.^2^ This may have contributed to the high levels of eosinophils in some of the NAR population in this study.

Higher eosinophil counts were associated with the increased risk of allergic subtype symptoms and the decreased risk of non-allergic subtypes in children (0–13 years).^26^ We observed a similar phenomenon in adolescents and adults (14 years and older), where patients with higher blood eosinophil levels were more likely to have comorbid asthma and allergic conjunctivitis. Using 0.3 × 10^9^/L as the threshold for blood eosinophil counts, the proportion of complicated asthma was significantly higher in the high-eosinophil group than in the low-level group. Rhinitis alone (local disease) and rhinitis with asthma multimorbidity (systemic disease) are considered to be 2 distinct diseases with significant differences in genomic and transcriptomic background, allergen sensitization patterns, symptom severity, and treatment response.^27^ A meta-analysis of studies including 133,813 participants showed that the overall prevalence of AR with comorbid asthma was 10.17%.^28^ In clinical trials, a single blood eosinophil count is frequently applied as a biomarker to identify the inflammatory phenotype in asthma patients and as an inclusion criterion for study entry.^29^ Several reports showed that blood eosinophil counts predict treatment outcomes with mepolizumab (IL-5 targeting therapy) use, especially in severe asthma, including the observation that with a baseline blood eosinophil count ≥0.15 × 10^9^/L, clinically meaningful reductions in exacerbation rates are achieved.^30^^,^^31^ Dupilumab, a fully human monoclonal antibody inhibits both IL-4 and IL-13 signaling, has been shown to be significantly effective in the treatment of subjects with persistent, moderate-to-severe asthma and a blood eosinophil count ≥0.3 × 10^9^/L or sputum eosinophils ≥3%.^32^ Eosinophils are involved in the inflammatory and regressive phases of the disease, therefore, focusing on changes in eosinophil levels is important for precision targeted therapy and efficacy prediction.

Extensive degranulation is a likely prerequisite for the pathogenic role of eosinophils. Previous studies have confirmed that seasonal allergen exposure is associated with exacerbated nasal symptoms, increased lavage fluid levels of ECP and α2-macroglobulin, and increased numbers of tissue eosinophils, as well as increased eosinophil degranulation and the production of large localized granulin deposits.^33^ Elevated eosinophils in patients with SAR with or without asthma during the pollen season compared with the non-pollen season was confirmed by sputum eosinophil counts and sputum ECP levels.^34^ However, blood and nasal secretions eosinophil counts are reliable and readily available biomarkers compared to nasal biopsies and sputum samples. To our knowledge, this is the first study to reveal an association between pollen exposure and eosinophil levels in blood and nasal secretions of patients with rhinitis, suggesting that systemic and local eosinophil levels can be used as indicators of sensitivity to SAR.

AR is a localized immediate reaction of the nasal mucosa in response to stimulation by inhaled allergens. Pal et al found that an eosinophil count >0.3 per HPF in nasal smears is a highly specific criterion for the diagnosis of AR.^35^ House dust mite nasal provocation test induces and aggravates both upper and lower airway inflammation and hyper-responsiveness in patients with PAR without asthmatic symptoms, and eosinophils in nasal lavage fluid increased significantly.^36^ Nasal secretions reflect the local inflammatory state of the nasal cavity and there is growing evidence for its potential role in the clinical diagnosis of allergy and the assessment of immunotherapy efficacy.^37^^,^^38^ Although nasal secretion eosinophil levels were less sensitive than blood eosinophils in identifying rhinitis subtypes and multimorbid allergic diseases in this study, the trend was consistent with systemic eosinophil levels, as well as being able to reflect the effects of seasonal allergen exposure and having its specificity for identifying patients with local AR (LAR). Overall, nasal secretion smear cytology, as a noninvasive test, is clinically important in the dynamic monitoring of the exposure-response relationship in SAR patients.

This study has its limitations. Firstly, this is a cross-sectional study based on a clinical phenomenon, and we are not aware of the eosinophil trends in AR patients over a longitudinal time series, so the exposure-response relationship between seasonal allergens and eosinophils will be further validated in a longitudinal cohort in the future. Secondly, we did not perform a nasal allergen provocation test (NAPT) to rule out the possible presence of LAR in the NAR population, which may be responsible for the significant difference in eosinophilic levels in nasal secretions of NAR patients during pollen versus non-pollen season. In addition, we excluded people who had used oral/topical drugs in the last week, which may have left a proportion of patients with severe symptomatic rhinitis unobserved. The efficacy of the drugs that play a role in this exposure-response relationship deserves to be explored in depth and may help to improve the approach and timing of allergy treatment. Finally, in this study, local eosinophil levels were graded instead of fully quantitative counting, which has limitations in identifying the difference in nasal local inflammatory status between people in pollen and non-pollen seasons, and may be the reason for the insignificant difference in local eosinophil levels in SAR patients.

## Conclusions

Our results found that blood eosinophils levels differed among NAR, PAR, and SAR patients, that overall nasal and ocular symptoms were more severe in patients with high blood eosinophils levels, and that blood eosinophils levels were significantly higher in patients with asthma or allergic conjunctivitis than in patients without comorbidities. There was a positive trend between allergen exposure and systemic and local eosinophil levels. Eosinophils are important in the diagnosis of allergic diseases, dynamic monitoring of allergen exposure, and screening of monoclonal antibody therapy.

## Abbreviations

CR, chronic rhinitis; AR, allergic rhinitis; NAR, non-allergic rhinitis; PAR, perennial AR; SAR, seasonal AR; ARIA, Allergic Rhinitis and its Impact on Asthma; sIgE, serum specific IgE; VAS, visual analogue scale; TNSS, total nasal symptoms; TOSS, total ocular symptoms; ATCS, average total combined score; ENR, eosinophil-neutrophil ratio; ELR, eosinophil-lymphocyte ratio; ECP, eosinophilic cation protein; EPX, eosinophil peroxidase; LAR, local AR; NAPT, nasal allergen provocation test.

## Funding

This work was supported by grants from Beijing Hospitals Authority Clinical medicine Development of special funding (ZLRK202303), national key R&D program of China (2022YFC2504100), the program for the Changjiang scholars and innovative research team (IRT13082), Natural Science Foundation of China (81970849, 82071022 and 82271141), CAMS innovation fund for medical sciences (2019-I2M-5–022), Beijing Municipal Science & Technology Commission (Z211100002921057 and Z211100002921060) and Capital's funds for health improvement and research (2022-1-1091).

## Availability of data and materials

All data generated or analyzed during this study are included in this published article.

## Authors’ contributions

All the authors contributed significantly to the study: XZ, LX and YZ collected the data. XX, YG and XL analyzed the data. XX and JL wrote the manuscript. YZ and LZ designed and supervised the study.

## Ethical approval

This study was approved by the ethics committee of Beijing Tongren Hospital of Capital Medical University (TREC2022-KY046). All patients provided informed consent, and the study protocol complied with the ethical guidelines of the Declaration of Helsinki.

## Authors’ consent for publication

All authors had final approval of the manuscript version to be published and are accountable for all aspects of the work in ensuring the accuracy and integrity of this manuscript.

## Declaration of competing interest

The authors have no relevant affiliations or financial involvement with any organization or entity with a financial interest in or financial conflict with the subject matter or materials discussed in the manuscript. This includes consultancies, employment, expert testimony, honoraria, speakers bureaus, retainers, stock options or ownership.
